# Predictors of Serious Psychological Distress in an Urban Population

**DOI:** 10.1007/s10597-014-9790-z

**Published:** 2014-12-23

**Authors:** Dritana Marko, Stephen H. Linder, Jessica M. Tullar, Thomas F. Reynolds, Larissa J. Estes

**Affiliations:** 1 Institute for Health Policy, School of Public Health, University of Texas Health Science Center at Houston, Houston, TX USA; 2 Texas Institute for Health Care Quality and Efficiency, Texas Health and Human Services Commission, Austin, TX USA

**Keywords:** Psychological distress, K6-scale, Mental health services, Community health, Health survey

## Abstract

While there are state and national estimates of serious psychological distress (SPD), these are not useful for targeting local mental health interventions or for addressing the needs of sub-populations at increased risk for SPD. This cross-sectional study uses data from the population-based 2010 Health of Houston Survey (n = 5,116) to examine (1) the prevalence of SPD and its determinants in Houston area and (2) predictors of the utilization of mental health services among people with SPD. The prevalence of SPD among the Houston area adult population was 7 %, more than twice the national average. Correlates of SPD included: being female, under 65, lacking emotional support, smoking, having poor health status and financial distress. The odds of utilizing health services by those with SPD were affected by financial distress, insurance, employment and perceived need for services, among other factors. Interventions should be tailored to mitigate risk factors for SPD and to improve access to mental health services in the SPD sub-population.

## Background

Mental illnesses make up the second-leading disease burden in the U.S. (WHO 2004), affecting more than 26 % of adults in a given year (Kessler et al. 2005). Mental health problems take a toll on society, as well as on the individual, and are often chronic, due in part to the lack of both early detection and access to preventive and treatment services (Haflin 2007). Serious mental illnesses can be manifested through symptoms of serious psychological distress (SPD), which is associated with poor social and emotional wellbeing, reduced quality of life (Shih and Simon 2008), higher health expenditures and healthcare utilization (Dismuke and Egede 2011) and is a predictor of physical illness (Egede and Dismuke 2012; Ferketich and Binkley 2005) and premature mortality (Huppert and Whittington 1995; Pratt 2009). Documenting mental illness in the general population can raise awareness among policymakers and provide for better targeting of services to those with unmet needs. However, relying on diagnostic procedures in clinical settings to identify mental health population-based needs is neither practical nor cost-effective. Consequently, screening tools for SPD are being implemented in general population surveys to estimate the prevalence of mental disorders.

The 2009 U.S. National Health Interview Survey (NHIS) found that 3.2 % of the adult population had experienced SPD (Reeves et al. 2011) with variations between states, even after adjusting for socio-demographic characteristics and insurance coverage. Texas showed higher (5.2 %) than national rates of SPD in 2007 (Strine et al. 2009b). These state and national estimates are of limited use for local planning, especially in diverse urban areas where SPD rates are higher than rural (Dhingra et al. 2009), and where there are substantial health disparities by race/ethnicity and income. Within states, many large urban areas (e.g., Houston), with high population density, are exposed to rapid changes in cultural and ethnic composition, and dramatic growth in the Hispanic population (Passel and Cohn 2008). Local information is essential for targeting mental health interventions and for addressing the needs of urban sub-populations at increased risk for psychological distress (Croft et al. 2009). Moreover, the SPD sub-population may be particularly vulnerable to factors related to access to much needed mental health services (e.g., insurance and financial barriers (Pearson et al. 2009), screening and referral system (McQuaid et al. 1999; Young et al. 2001), recognition of the need for services, stigmatization (Sirey et al. 2001; Thornicroft 2008; Thornicroft et al. 2008; USDHHS 1999, 2003), negative beliefs about mental illness (Segal et al. 2005) and knowledge of mental health services (Mickus et al. 2000).

The aim of this study is to estimate the prevalence, and the associated factors, of SPD in adult population, in a large urban area such as Houston. In addition, we examine the predictors of access to mental health services within the SPD sub-population.

## Methods

### Data Collection

Data are drawn from the 2010 Health of Houston Survey (HHS), a complex, stratified random sample survey of non-institutional households in Harris County and the City of Houston conducted from November 2010 to March 2011. Details of the survey’s methodology and questionnaires can be found at www.hhs2010.net. HHS was designed to collect comprehensive information on the state of health, prevalence of chronic diseases and mental health, insurance coverage, access to care and socioeconomic and demographic characteristics. Sampling strata were designed to be representative of the adult population across seven sub-county areas, defined by the U.S. Census as 1 % Public Use Microdata Areas (1 % ACS-PUMAs), as well as gender, race and ethnic groups, age grouping and poverty levels. To ensure maximum coverage, households were randomly selected from residential addresses contained in the U.S. Postal Service Delivery Sequence File (DSF). This method captures households with landline phones, cell phone‐only [24.9 % in the U.S. (Blumberg et al. 2011) and 32.4 % in the Houston area (HHS 2011)] and non‐phone households (about 2 % of all households). The “Rizzo” method (Rizzo et al. 2004) was used to randomly select respondents within households. Nonrespondents were replaced with respondents with similar characteristics to achieve the required sample and group representativeness. Informed consent from the participants was obtained verbally at the beginning of the interview. Survey design and methods were reviewed and approved by the University of Texas Health Institutional Review Board.

To attain the highest completion rate possible, HHS was administered via computer-assisted telephone interviewing (CATI), online, and by mail, in English, Spanish and Vietnamese. Questions were drawn primarily from other federal surveys (e.g., National Health Interview Survey and the Behavioral Risk Factor Surveillance System). To maximize cooperation and response rate, an invitation letter with a $2 incentive was sent to the selected households, followed by several rounds of reminders when needed. Using standard definitions (AAPOR 2010), the cooperation interview rate was 66 % and the final overall response rate 29 %. The final sample was much larger (n = 5,116) than the estimated required sample (n = 4,000).

Overall we excluded 89 participants who responded “don’t know” or “refused” in all SPD items, 374 respondents were excluded from the SPD analysis and 97 from the mental health visit analysis. There were some differences between the excluded and the included (e.g., in the overall SPD model the excluded were better off on income-related measures than their counterparts) but the pattern of differences across models was inconsistent.

### Serious Psychological Distress

To assess SPD, we used the Kessler 6-item tool, validated nationally and internationally (Kessler et al. 2010) and used in other national surveys (e.g., the National Health Interview Survey, the National Survey on Drug Use and Health), that screens for the presence of serious mental illnesses in the general population (Kessler et al. 2002; Kessler et al. 2003). Respondents are asked about the frequency of feeling nervous, hopeless, restless or fidgety, sad or depressed, that everything was an effort, and worthless in the last 30 days. Consistent with standard practice on federal surveys, if a single SPD item was missing, the imputed value was used. Missing values were imputed using a multiple imputation technique (Little and Rubin 2002). Responses vary on a 5-point Likert scale scored from 0 (never) to 4 (always) for a composite score from 0 to 24, where 13 or higher indicate presence of SPD.

### Mental Health Services Utilization Among People with SPD

Mental health visits were assessed with two questions to create two separate outcomes. The first question was a K6 scale follow-up question (i.e., “During the past 30 days, how many times did you see a doctor or other health professional about these feelings?”). Those who reported “1 or more times” were coded as having used mental health services in the last month. Although the K6 scale focuses on the last 30 days for recall purposes, serious mental health conditions are typically chronic and symptoms appear prior to full clinical manifestation. Thus, assuming that those exceeding the SPD threshold were likely to have had experienced symptom onset at some time in the past and may have sought services, we asked if “In the past 12 months, have you seen your doctor or other professional for problems with your mental health, emotions, nerves or use of alcohol or drugs?”. Those saying “yes” to visits in the last 12 months or in the last 30 days were coded as having had mental health visits in the last 12 months.

### Determinants of SPD and of Mental Health Services Utilization Among People with SPD

Based on the available literature, we identified six categories of individual and community-level variables relevant to the risk of SPD. (1) *Demographic characteristics* include gender, age, race/ethnicity, marital status, immigration status, non-English linguistic isolation and insurance status. Respondents were considered uninsured if they answered “no” to all the insurance options offered (i.e., employer-sponsored, self-purchased, Medicare, Medicaid, CHIP, TRICARE, CHAMPUS, CHAMP-VA, VA or other plan). (2) *Socio-economic standing* includes: educational attainment, employment, household income measured by the Federal Poverty Level (FPL), personal income before taxes, and home ownership. (3) *Financial stressors and economic hardship* were measured by two questions asking about the frequency of having had financial difficulty in the last year buying food or paying for utilities, or making rent or mortgage payments (responses “sometimes”, “often” or “always” were coded as having had such difficulty), a yes–no question on experiencing delay or inability to fill prescriptions due to their cost or a lack of insurance, and a fourth yes–no question on difficulties paying medical bills in the last 12 months. Economic hardship questions were not asked to those with household income of $150,000 or greater; these (n = 358) were coded as “no hardship.” Total household members and number of children in the household were included as possible contributors to financial distress. (4) *Social support* included three items (i.e., tangible social support, positive social interaction and emotional/informational support) from the Social Support Questionnaire of the Medical Outcome Study (Sherbourne and Stewart 1991). (5) *Health status* was measured by self-reported general health status, at least one diagnosed chronic condition or disease (such as diabetes, heart attack, stroke, ischemia, hypertension, asthma, and cancer), obesity, calculated from self-reported weight and height (Body Mass Index ≥ 30), and current smoking. (6) *Neighborhood stressors* were measured by a composite variable intended to reflect neighborhood problems: concern with lack of the availability of fresh fruits and vegetables; crime; stray animals; water pollution from harmful chemicals and run off; polluted drinking water; waste dumping; traffic, and industrial pollution. The scores varied from 0 to 8, depending on the number of factors reported to be a neighborhood problem.

Finally, and exclusively for the analysis of the determinants of mental health services utilization among people with SPD, we added a proxy for perceived mental health need (i.e., “Was there ever a time during the past 12 months when you felt that you might need to see a doctor or other professional because of problems with your mental health, emotions, nerves, or your use of alcohol or drugs?”).

### Statistical Analysis

We used logistic regression to estimate the association (odds ratio [OR] and corresponding 95 % confidence interval [95 % CI]) between the potential determinants and each outcome. The strategy to select variables for inclusion in our models followed standard recommendations (Hosmer and Lemeshow 2000). First, we examined the Wald test *p* value of the bivariate associations between each determinant and SPD. Variables with *p* < 0.25 were included in subsequent models. Next, separate models were run within each set of determinants (i.e., socio-economic disadvantage proxy indicators, health disparities proxy indicators, financial, neighborhood stressors, social support and health status). Variables with *p* < 0.10 within each set were included in a multivariate model. The final model included only variables with *p* < 0.05 using a backward elimination procedure. For the mental health visits models (1 in the last year, and 2 in the last month), a similar procedure was used but given the smaller sample size for these analyses, we used a less restrictive *p* value (*p* < 0.10) in the final multivariate model.

Final models were checked for multicollinearity which was not found to be a concern (tolerance > 0.1). The area under the Receiver Operating Characteristic (ROC) curve, which reflects the power of each model to discriminate between cases and non-cases (Heeringa et al. 2010), for all final models was satisfactory (0.85 for the SPD model, 0.82 for mental health visits in the last year model and 0.76 for mental health visits in the last month model). Analyses were conducted in Stata^©^ (StataCorp. 2011), accounting for the complex sampling design of the survey. Data were weighted to represent the county’s demographic characteristics based in 2010 U.S. Census and to reduce the effect of nonresponse and sampling coverage gaps on the reliability of the survey results (Groves 2006; Keeter et al. 2000).

## Results

The prevalence of SPD was 7 %, 56.9 % of which did not have mental health visits in the last 12 months and 70.3 % did not have any mental visits during the last 30 days. The study population had slightly more females (51 %), and 45 % aged 18–39 and 40–64. Nearly 50 % had a high school (HS) diploma or less education; 63 % were married or living with a partner; 32 % were immigrants; and 16 % were linguistically isolated. White and Hispanic sub-populations were about equal in size. Approximately 32 % were living at or below the FPL. A seventh of the study population was unemployed and looking for work. More than a third did not own their place of residence; nearly 43 % were living in households with at least four people; and more than half lived in households with no children. Compared to the overall population, the group who scored positive for SPD was mostly female (68 %), aged 40–64 (61 %), with a HS education or less (66 %) and of Hispanic ethnicity (38 %). It also included a higher proportion of African-American residents (27 vs. 17 %), more uninsured (40 vs. 31 %), with less education (66 vs. 49 %), more unemployed (28 vs. 14 %) and proportionately more considered poor by FPL (50 vs. 32 %).

The characteristics that were the strongest predictors of SPD were: female (OR 2.0; 95 % CI 1.3–3.0), under 65 (OR 2.7; 95 % CI 1.5–4.8 for ages 40–64; OR 1.9; 95 % CI 1.1–3.6 for ages 18–39), lacking emotional support (OR 2.6; 95 % CI 1.8–3.8), smoking (OR 1.8; 95 % CI 1.2–2.8), and having poor health status (OR 2.8; 95 % CI 2.0–4.1). Difficulty paying rent or mortgage (OR 2.4; 95 % CI 1.6–3.5), difficulty paying for medical bills (OR 1.8; 95 % CI 1.2–2.7) or prescriptions (OR 1.9; 95 % CI 1.2–1.9), and living in a neighborhood with problems (OR 1.1; 95 % CI 1.0–1.2), also increased the likelihood of SPD. Living in a household with one child reduced the likelihood of SPD (OR 0.4; 95 % CI 0.2–0.7), compared to those living households with no children.

Within the SPD group, the likelihood of using mental health services depended on multiple factors. In the final multivariate models, females (OR 2.9; 95 % CI 1.2–7.1), those having problems paying for medical bills (OR 3.0; 95 % CI 1.3–7.0), those unemployed and not looking for work (OR 3.1; 95 % CI 1.3–7.4) and those expressing a perceived need for mental health services (OR 3.7; 95 % CI 1.6–8.9) were the most likely to have used mental health services in the last year. Conversely, being uninsured (OR 0.2; 95 % CI 0.1–0.5) and having low emotional support (OR 0.4; 95 % CI 0.2–0.8) were among the least likely to have used mental health services in the last year. The characteristics of those most likely to have completed visits for mental health services in the past month included: living under the Federal Poverty Level (OR 2.7; 95 % CI 1.1–6.6) and reporting poor health (OR 2.3; 95 % CI 1.1–5.0). Uninsured (OR 0.2; 95 % CI 0.1–0.4) and non-White members of the SPD group were least likely to have had visits in the past 30 days.

## Discussion

Our findings show that the prevalence of SPD in a large urban area, such as the Houston area, was substantially higher than either the corresponding state or national rates and that among those with SPD (with presumably a higher need for services) more than half reported not having received any mental health care. Our study also showed that residents exposed to financial stressors (i.e., difficulty paying rent, mortgage, medical bills, or filling prescriptions) and a lack of emotional support had significantly higher prevalence of SPD, in contrast to those unaffected by those stressors and being able to count on emotional support. Finally, being female, having difficulty paying medical bills and reporting the need for mental health care were associated with a greater likelihood of actually obtaining mental health services.

The role of financial hardship, as opposed to personal or household income, has been previously suggested (Short 2005) and appears as a key risk factor for adverse health outcomes (Ahnquist et al. 2007; Laaksonen et al. 2007; Weich and Lewis 1998). Although income and poverty level were significantly related to SPD in the bivariate models, this relationship did not hold up in the multivariate analysis. Other studies (Chittleborough et al. 2011; McVeigh et al. 2006) have found income to be an independent predictor of SPD. A possible explanation might be the failure to include economic hardship indicators in these studies or to account for assets, as well as financial and medical debt. Other studies have confirmed the association of financial stressors with selected mental disorders, adjusting for other factors, but not with low levels of income or SPD (Laaksonen et al. 2007; Wang et al. 2010).

Although we assessed three kinds of social support, only emotional/informational support appeared to be relevant, which agrees with other studies that have found emotional support to be the most helpful buffer against stressful situations (Cohen 2004). Even the people who report only instrumental support attach emotional meaning to this support (Semmer et al. 2008). Our finding regarding the negative effect of the lack of emotional/informational support is consistent with the literature showing an association between lack of social support and psychological distress (and mental health problems more generally) (Andrews et al. 1978; Cohen et al. 2000).

While other results in our study (e.g., increased SPD prevalence among ages 18–64, women, those having poor health status and smokers) are consistent with previous national studies (Kessler et al. 2010; Reeves et al. 2011; Strine et al. 2009a), some of the indicators that have previously been associated with SPD were not relevant in our final model. For instance, race/ethnicity, frequently reported as important, did not seem to play a role in the prevalence of SPD, given the ethnic and racial diversity of the urban setting in our study. It has been argued by others that different racial/ethnic and linguistic groups appear to report mental health needs in different ways and, more importantly, that racial and ethnic differences diminish when models adjust for income and other socio-economic factors (Grant et al. 2010). Conversely, in our study the effect of income was concentrated at the lowest level only and was not responsible for the lack of differences among race/ethnicity groups. Further research will be needed to corroborate our results.

Regarding the use of services, the finding that females are more likely to have received mental health services, mirrors the disproportionate prevalence of SPD among females. The relationship between difficulty paying medical bills and greater likelihood of receiving services may reflect a connection between debt and mental health service use. Lack of insurance coverage increases the likelihood of no services (and unmet needs); this agrees with the previous literature as the principal reason for lack of service use among those in need (Mojtabai 2009; SAMHSA 2012). The finding on the reduced likelihood of using services among those lacking emotional support is reasonable since support may enhance recognition of service needs and can countervail against cultural stigmas (Thornicroft 2008) that undervalue services or suppress recognition of mental health conditions.

Overall, discrepancies between previous studies and the current one could be related to differences in geography, survey designs or sample sizes. The cross-sectional design of our study precludes establishing causality or directionality of the associations. Further, measures in our study were self-reported and, as with all survey designs, are subject to recall bias and potential social desirability effects. While the address-based sampling used in the HHS makes response rates across non-address-based designs difficult to compare, address-based sample provides an appropriate approach for excellent coverage of the non-institutionalized population since corrections for non-coverage are difficult to implement from the sample itself (Kish 1990). The use of complex random stratified sampling strategy makes the sample representative of the current population in the Houston area, and methods such as weighting and imputation, correct for potential biases of selection, of variance and of nonresponse. Moreover, we obtained 25 % more sample than the initially target sample.

Our data show that the presence of SPD increases the likelihood that mental health services will be sought. The extent to which these services are obtained, however, will depend not only upon recognition of need and, in turn, on the severity of symptoms and resulting impairment, but also on certain enabling factors that affect chances of successful access, such as insurance coverage and employment status (Bebbington et al. 2000; ten Have et al. 2001). Since the K-6 items are thought to tap into enduring conditions (Huppert and Whittington 1995; Kessler et al. 2010), we included mental health visits for up to 12 months in the past, as well as an independent measure for the last 30 days. Using visits in the last 12 months errs on the side of overestimating visits for those with recall bias, and thus counters a tendency to overstate unmet needs for SPD cases without visits.

## Conclusions

The present study adds to the current knowledge of risk factors for SPD with data drawn from an urban area. To our knowledge, only one other study considers SPD in an urban setting (McVeigh et al. 2006), and none have examined perceived unmet needs among those with SPD. We expand upon the previous work by introducing a wide variety of financial stressors and demographic indicators, proven to be associated with health disparities and socio-economic disadvantage. We also add psycho-social factors, neighborhood-level stressors, and risk behaviors, such as smoking and obesity. Our findings suggest that strategies and policies could be better tailored to those population groups whose characteristics appear to be associated with the highest likelihood of SPD. Finally, greater attention should be given to people with SPD who need mental health services but are unable to access them. Our results suggest that this group includes the uninsured and those with low emotional support. These results can be expected to apply to other urban areas with similar demographic profiles, including a growing percentage of people of Hispanic origin, dealing with financial distress, particularly in the south and southwest regions of the U.S.
